# Advancing patient-centred care in clinical trials

**DOI:** 10.1016/j.lanwpc.2025.101569

**Published:** 2025-04-30

**Authors:** The Lancet Regional Health – Western Pacific

The Asia–Pacific region accounts for more than half of all global clinical trials activity and the number is expected to continue expanding, driven by the increasing incidence of cancer, a large patient population, relatively low cost, considerable support from the government, and the presence of top clinical trial centres. However, patient-centric trials are still a relatively new concept in the region.

Patient-centric trials designed and conducted with patients' needs, preferences, and experiences as the focal point will shape the future of clinical trials, which can improve patient experiences and amplify patient health gains with holistic care and overall wellness rather than solely concentrating on disease management. Balancing the interests of industries, scientific integrity, and patient preferences during trial design can be complex, and patients' needs may vary significantly due to individual, cultural, and demographic factors. Cultural and language diversity impact patients' understanding of trial information, fragmented regulations lead to unpublished or selectively reported results, and lack of trust in investigational drugs and limited awareness and knowledge of clinical trials hamper patients’ engagement in clinical trials. Including the plan to report trial results to patients in the study protocol and developing a context-specific communication model, adopting international transparency standards (eg, WHO guidelines), empowering patients with fundamental knowledge about the disease and the concept of clinical trials through localised educational workshops, as well as collaborations with patient advocacy groups, may help to address these obstacles.

To improve the implementation of patient-centric trials in the Asia–Pacific region, we need to consider additional elements that may be more readily attainable and significantly contribute to the transition towards patient-centricity.

The first such improvement could be to rethink recruitment before the trial to facilitate more flexible eligible criteria and equitable inclusion to reflect the real-world setting. Inclusion criteria for clinical trials are always strict, with the majority of patients not meeting the enrolment criteria. While a stringent criterion aims to ensure the homogeneity of trial participants to reduce variability that could confound results, this may not accurately reflect the effectiveness and safety of treatments for patients who will ultimately receive the drugs in real-world settings. In 2019, the US Food and Drug Administration issued new recommendations for broadening cancer trial eligibility criteria, to promote inclusion of paediatric patients and patients with comorbidities (such as patients with HIV, hepatitis B and C virus infections, brain metastasis, or organ dysfunction). Countries in the Asia–Pacific region should also work to align clinical trial eligibility criteria with more inclusive international standards. This could maximise the generalisability of trial results; however, the potential adverse impacts of expanding enrolment—patient safety, regulatory compliance, integrity and reliability of trial findings, and possible economic loss for industries—need careful consideration.

Second, patients’ voices can be amplified during clinical trials by adopting adaptive trial designs and reporting patient-reported outcomes (PROs). Traditional trials primarily prioritise scientific and regulatory requirements and require stringent compliance during the trial. Adopting adaptive trial designs that allow re-examining and adjusting study protocols (such as dropping a treatment group for lack of efficiency or focusing on certain subgroups) based on interim data can minimise the harms and maximise the practical value of patients. However, the methodological and operational complexity of adaptive trials poses additional challenges in statistical analysis, ethical considerations, and funding. Incorporating PROs into oncology trials is essential, especially as patients with cancer now have longer survival and want to ensure the best possible quality of life throughout the cancer journey. Health-care stakeholders have recognised the importance of PROs. In 2023, the China National Medical Products Administration released three technical guidelines for clinical trials focused on a patient-centred approach for the first time, and PROs were one of the major factors. Nevertheless, there are currently insufficient standard measurement and reporting of PROs, as well as the interpretation of PRO data and potential conflicts between clinical outcomes and PROs. In a study of cancer drugs approved in China, it was found that fewer than one-third of cancer indications approved between 2005 and 2020 had shown health-related quality of life (HRQoL) improvements, with considerable heterogeneity in the analysis and reporting of HRQoL benefits. This emphasises the necessity of including evaluation and analysis of PROs in clinical research and improving the standardisation of HRQoL assessment. PRO measurements are also important for accurate cost-effectiveness studies for expensive cancer indications—without PRO data collected during trial, the quality-adjusted life years estimated during the economic modelling can be adapted from literature and prone to bias.

Despite the challenges, it's encouraging to see governments and pharmaceutical companies start to build partnerships with patient advocacy groups to collaboratively develop trial designs. The development of new trial models facilitates the implementation of patient-centred trials. A small yet innovative patient-centric trial exemplifies how we can effectively prioritise and enhance patient engagement. This adaptive umbrella trial consists of a criteria-fulfilled cohort, a compassionate-use cohort for patients who do not meet the inclusion criteria, and, for patients who were unwilling to be enrolled in the trial for frequent follow-up, a real-word study cohort who received routine treatment (ie, physician's choice of treatment) to observe clinical outcomes. Through such cohort design, the effectiveness of the drug in the eligible patients and a broader patient population, as well as its comparison to real-word clinical practice, can be determined. This also leads to better treatment access and early evidence to support faster regulatory approval for on-label use among patients with comorbidities. Decentralised clinical trials (DCTs) utilise digital health technologies, including wearable devices, telemedicine, or mobile applications for consent, recruitment, screening, and remote monitoring, to improving accessibility of clinical trials. Although DCTs can minimise the burden on patients during participation in clinical trials and widen access to previously underserved populations, potential costs of digital technologies from the researcher and industry perspective may be another challenge. Additionally, a study presented in this year's Japanese Society of Medical Oncology Annual Meeting (JSMO 2025) suggested that DCTs have increased the workload for the responsible clinical research coordinators and project managers.

With the growth of attention on patient-centric trials in the Asia–Pacific region, *The Lancet Regional Health*—*Western Pacific* calls for clinical trials to maximise benefits for patients and adopt more flexible inclusion criteria, report PROs, and employ adaptive trial designs. We encourage content on patient-centred trials as well as real-word data to demonstrate the practical value patients experience from clinical trials.

